# Effects of Graded Levels of Sunflower Hulls in Diets of Naemi Ewes During Late Gestation and Lactation on Colostrum Quality, Milk Composition, Lamb Performance, Survival, and Blood Metabolites

**DOI:** 10.3390/vetsci13070694

**Published:** 2026-07-17

**Authors:** Mohsen M. Alobre, Ibrahim A. Alhidary, Mohammed M. Qaid, Abdulkareem M. Matar, Hani H. Al-Baadani, Abdulrahman S. Alharthi, Ahmad A. Aboragah, Ahmed A. Alghonaim, Riyadh S. Aljumaah, Mutassim M. Abdelrahman

**Affiliations:** Department of Animal Production, College of Food and Agriculture Sciences, King Saud University, Riyadh 11451, Saudi Arabia; ialhidary@ksu.edu.sa (I.A.A.); abdmatar@ksu.edu.sa (A.M.M.); hsaeed@ksu.edu.sa (H.H.A.-B.); abalharthi@ksu.edu.sa (A.S.A.); aaboragah@ksu.edu.sa (A.A.A.); ahghonaim@ksu.edu.sa (A.A.A.); rjumaah@ksu.edu.sa (R.S.A.); amutassim@ksu.edu.sa (M.M.A.)

**Keywords:** colostrum quality, lamb growth performance, maternal nutrition, Naemi sheep, neonatal survival, sunflower hulls, sustainable feeding

## Abstract

(1) Background: Sunflower hulls (SFHs) are an abundant agro-industrial by-product that may serve as an alternative fiber source in sheep diets, particularly in arid production systems. This study evaluated the effects of graded levels of dietary SFH during late gestation and lactation on colostrum and milk composition, lamb growth performance, neonatal survival, and serum biochemical parameters in Naemi sheep. (2) Methods: Seventy-two primiparous Naemi ewes (initial body weight = 55.0 ± 3.8 kg) were randomly allocated to four dietary treatments containing 0, 12, 20, or 28% SFH (*n* = 18 ewes per treatment) from late gestation until lamb weaning at 90 days postpartum. Data were analyzed using mixed-model procedures, and treatment effects were considered significant at *p* ≤ 0.05. (3) Results: Dietary SFH supplementation did not affect lamb birth weight or overall survival rate (*p* > 0.05). However, weaning weight declined from 28.03 kg in the control group to 25.11 kg in the 20% SFH group, showing a significant linear decrease with increasing SFH inclusion (*p* = 0.044). Likewise, average daily gain decreased linearly by 10.4% (*p* = 0.044). Colostrum composition was partially affected by dietary treatment, with fat and protein concentrations at 48 h postpartum increasing quadratically by 46.6% and 33.50%, respectively, in ewes fed 20% SFH compared with the control group (*p* < 0.05). In contrast, milk fat and total solids contents decreased quadratically by 30.7% and 13.4%, respectively, in ewes receiving 12% SFH (*p* = 0.01). Most serum metabolite variables were unaffected by dietary treatment and remained within normal physiological reference ranges. However, overall serum triglyceride concentration decreased linearly with increasing SFH inclusion (*p* = 0.023). (4) In conclusion, SFH can be incorporated into the diets of Naemi ewes during late gestation and lactation as an alternative fiber source without adversely affecting lamb birth weight, survival, or overall metabolic health. However, increasing dietary SFH inclusion reduced pre-weaning lamb growth despite improvements in selected colostrum quality traits. Based on the productive and physiological responses observed, 12% dietary SFH appears to be the optimal inclusion level, providing the best balance between animal performance and sustainable feed utilization.

## Simple Summary

Sunflower hulls are an abundant agricultural by-product that could help reduce feed costs and improve the sustainability of sheep production, particularly in arid regions where conventional feed resources are limited. This study investigated whether adding different amounts of sunflower hulls to the diets of Naemi ewes would affect the quality of colostrum (the first milk produced after birth), milk composition, lamb growth, survival, and health. Seventy-two ewes were fed diets containing different levels of sunflower hulls from late pregnancy through lactation. Feeding sunflower hulls did not affect lamb birth weight or survival, indicating that maternal supplementation did not impair fetal development or neonatal viability. However, increasing dietary sunflower hull inclusion reduced pre-weaning lamb growth, although some improvements in colostrum fat and protein concentrations were observed. Blood biochemical measurements remained within normal physiological ranges, indicating that lamb metabolic health was maintained. Overall, these findings suggest that sunflower hulls can be used as an alternative and sustainable fiber source in diets for pregnant and lactating ewes, provided that dietary inclusion levels are carefully managed to maintain optimal lamb growth and productivity.

## 1. Introduction

Maternal nutrition during late gestation and early lactation is a major determinant of reproductive efficiency, colostrum quality, milk production, lamb survival, and postnatal growth performance in sheep [1,2]. During these physiological stages, nutrient requirements increase substantially to support rapid fetal growth, mammary gland development, and milk synthesis [2]. Inadequate nutrient supply can impair fetal development, reduce passive transfer of immunity, compromise neonatal viability, and ultimately decrease lamb growth and survival [3,4,5,6]. In arid and semi-arid regions such as Saudi Arabia, sheep production is further challenged by the high cost and seasonal scarcity of conventional feed resources, highlighting the need for sustainable and economically viable alternative feed ingredients [7]. Consequently, the use of locally available agro-industrial by-products has attracted increasing attention as a strategy to reduce feeding costs while improving the environmental and economic sustainability of livestock production through more efficient resource utilization and reduced dependence on conventional feedstuffs [8,9].

Among the available agro-industrial by-products, sunflower hulls (SFHs), a residue of the sunflower oil industry, have emerged as a promising alternative fiber source because of their wide availability, low cost, and high structural carbohydrate content [10]. Dietary fiber is essential for maintaining rumen function by stimulating chewing activity, saliva secretion, and normal fermentation patterns [11]. However, the relatively high lignin content of SFH may reduce nutrient digestibility and dietary energy availability when included at excessive levels [10,12,13,14]. Consequently, identifying appropriate inclusion levels is essential to maximize the nutritional and economic benefits of SFH while minimizing potential adverse effects on animal productivity. Excessive inclusion of poorly digestible fiber may reduce feed efficiency and energy utilization, leading to lower milk production, altered milk composition, and reduced offspring growth [15,16,17]. Maternal nutrition also influences colostrum composition, particularly fat and protein concentrations, which are critical for neonatal energy supply, passive immunity, thermoregulation, and early-life survival [18]. Furthermore, blood metabolites such as glucose, cholesterol, triglycerides, total protein, and albumin are widely recognized as indicators of nutritional status, metabolic adaptation, and health in growing lambs [19,20], providing valuable insights into the physiological responses of offspring to maternal dietary interventions.

Previous studies have shown that dietary SFH can influence nutrient utilization, ruminal fermentation, colostrum quality, and animal performance [21,22]. In Naemi ewes, dietary inclusion of 12–28% SFH increased dry matter intake without affecting body weight or the digestibility of most nutrients, although higher inclusion levels reduced total volatile fatty acid production and altered ruminal fermentation characteristics [22]. Similarly, dietary SFH increased colostrum fat concentration and improved the colostral fatty acid profile, particularly at inclusion levels of 12–20%, whereas higher levels negatively affected some fatty acid fractions, likely because of increased lignin intake [21]. In growing Barki lambs, sunflower-derived feed ingredients improved nutrient digestibility, nitrogen retention, feed efficiency, growth performance, and several blood biochemical indices while reducing serum cholesterol and triglyceride concentrations [23]. More recently, complete replacement of wheat straw with SFH in pelleted diets for Naemi ewes maintained metabolic health but reduced lamb survival and reproductive performance compared with soybean hulls, indicating that the response to alternative fiber sources depends on both their nutritional characteristics and the physiological stage of the animals [24].

Although previous studies demonstrate the nutritional potential of SFH, evidence remains limited regarding the effects of graded dietary SFH inclusion during the critical transition period from late gestation to early lactation on colostrum and milk composition, lamb growth, neonatal survival, and metabolic responses in Naemi ewes raised under arid production conditions. Furthermore, the dietary inclusion level that optimizes the benefits of SFH while avoiding the negative effects associated with excessive lignified fiber has not been established. We hypothesized that moderate dietary inclusion of SFH would provide sufficient physically effective fiber to support maternal and offspring performance without adversely affecting metabolic health, whereas higher inclusion levels would reduce productive performance because of lower nutrient availability. Therefore, the objective of this study was to evaluate the effects of graded dietary inclusion levels of SFH in diets for Naemi ewes during late gestation and lactation on colostrum and milk composition, lamb growth performance, lamb survival, and selected blood metabolites under arid environmental conditions.

## 2. Materials and Methods

### 2.1. Animal Welfare and Ethics Clearance

The experiment was conducted at the experimental station of King Saud University (KSU), Riyadh, Saudi Arabia. All animal handling and experimental procedures were performed in accordance with the Animal Welfare Act and the Guidelines for the Care and Use of Animals for Scientific Purposes. The study protocol was approved by the Research Ethics Committee of King Saud University (REC-KSU; Ethics Reference No. KSU-SE-20-27).

### 2.2. Animals, Experimental Design, and Dietary Treatments

A total of 72 primiparous Naemi ewes (body weight, 55.0 ± 3.8 kg; 9–10 months of age) were enrolled during late gestation (approximately 90 days before lambing) and followed through lactation until 90 days postpartum. Pregnancy was confirmed by ultrasonography before the start of the experiment, and all animals were monitored throughout gestation, lambing, and lactation by a specialist veterinarian. The ewes were obtained from the KSU experimental station and were individually identified using ear tags.

At the start of the experiment, each ewe was weighed and assigned a BCS. Animals were then stratified according to initial body weight and BCS to ensure comparable baseline characteristics among treatment groups. Within each stratum, ewes were randomly assigned to one of four dietary treatments (0, 12, 20, or 28% SFH) using a completely randomized design, resulting in 18 ewes per treatment. Random allocation was performed using a computer-generated randomization procedure to minimize selection bias and ensure balanced distribution of animals among treatments.

Ewes were housed in shaded pens (6.5 × 6.0 m), with six animals per pen and three replicate pens per treatment. Each pen was equipped with feeding and watering facilities. Before the experimental period, all animals underwent a 14-day adaptation period during which they received a common basal diet formulated to meet maintenance requirements equivalent to 2% of body weight [4]. Following adaptation, ewes were assigned to one of the following dietary treatments:

C: Basal pelleted complete diet without sunflower hulls (control).
S12: Basal diet containing 12% SFH.
S20: Basal diet containing 20% SFH.
S28: Basal diet containing 28% SFH.

The selected dietary inclusion levels of SFH (12%, 20%, and 28% of dietary DM) were based on previous studies in Naemi sheep demonstrating favorable responses at moderate inclusion levels (12–20%) and potential reductions in nutrient utilization and ruminal fermentation at higher inclusion levels (28%) [21,22]. Rather than using equally spaced inclusion levels (e.g., 10, 20, and 30%), these concentrations were selected to evaluate biologically relevant thresholds under practical feeding conditions and to identify the inclusion level that optimizes productive performance while minimizing the adverse effects associated with excessive lignified fiber.

All experimental diets were formulated as complete pelleted total mixed rations to meet the nutrient requirements of ewes during late gestation and lactation according to NRC [4] recommendations (Table 1). Feed was offered *ad libitum* twice daily, and fresh drinking water was available continuously. The feeding trial extended from approximately 90 days prepartum until 90 days postpartum. Throughout the experiment, animals were monitored daily for health status, feed consumption, behavior, and any signs of illness or distress. No adverse health events were observed, and all ewes remained clinically healthy throughout the study.

Before lambing, individual lambing pens were cleaned, disinfected, bedded with fresh straw, and equipped with feed and water facilities to provide a clean and comfortable environment for parturition and neonatal care. Ewes showing signs of imminent parturition were transferred to these pens and remained there with their lambs for five days after lambing to facilitate neonatal management and sample collection. Lamb birth weight was recorded immediately after birth, and colostrum samples were collected from the corresponding dams.

After the neonatal period, ewe–lamb pairs were returned to their original treatment pens and remained together throughout the suckling period, where the lambs remained with their dams and suckled naturally throughout the suckling period. Lamb body weight and survival rate were recorded from birth until weaning at 90 days postpartum.

The BCS was used to evaluate the nutritional status of the ewes during late gestation (30 days prepartum) and early lactation (30 days postpartum). BCS was assessed in a representative subset of 48 ewes (12 ewes per treatment) selected from the total population of 72 ewes using the dorsal-palpation technique described by Santucci et al. [25]. Scores were assigned on a 0–5 scale with 0.5 increments. This subsampling approach was adopted because BCS assessment is a subjective procedure requiring individual animal handling and evaluation by trained personnel; therefore, evaluating a representative subset of animals per treatment was considered sufficient to characterize treatment effects while minimizing handling time and animal disturbance. In addition, BCS records from several ewes were excluded from the analysis because the measurements were incomplete or could not be verified with sufficient confidence to ensure data quality and consistency. Ewes were subsequently classified into four BCS categories (≤2.0, 2.5, 3.0, and ≥3.5).

### 2.3. Lamb Growth Performance, Survival, and Health Status

Lambs born to ewes in each treatment group were weighed at birth and subsequently at 30, 60 and 90 days of age using a calibrated digital livestock scale. Body weight gain (BWG) and average daily gain (ADG) were calculated for each lamb [26].

Average daily gain was calculated as follows:

$$ADG\;=\frac{Final\;body\;weight\;-\;Initial\;body\;weight}{Number\;of\;days}\;$$

Lamb health was monitored daily throughout the experimental period by veterinary personnel. Mortality events were recorded as they occurred, and the survival rate at weaning was calculated using the following equation as described by [27]:

$$Lamb\;survival\;rate\left(\%\right)=\frac{Number\;of\;lambs\;alive\;at\;weaning}{Total\;number\;of\;lambs\;born}\;\times \;100\;$$

### 2.4. Blood Collection and Biochemical Analysis

Blood samples were collected from all lambs by jugular venipuncture on day 1 after birth and subsequently at monthly intervals until weaning [28]. At each sampling, approximately 10 mL of blood was collected into plain vacutainer tubes (BD Vacutainer^®^, Franklin Lakes, NJ, USA). Samples were centrifuged at 2400× *g* for 15 min at 4 °C, and the serum was separated and stored at −20 °C until analysis.

Serum concentrations of glucose, total protein, albumin, urea nitrogen, total cholesterol, triglyceride, and non-esterified fatty acids (NEFAs) were determined using commercial diagnostic kits according to the manufacturers’ instructions and measured with a semi-automated analyzer (Randox Laboratories, Ltd., Crumlin, UK) [24]. Serum globulin concentration was calculated as the difference between total protein and albumin [29].

Total protein was determined using the biuret method, albumin by the bromocresol green dye-binding method [30], glucose by the glucose oxidase–peroxidase (GOD–POD) method [31], urea nitrogen by the urease–glutamate dehydrogenase enzymatic method [32], total cholesterol by the cholesterol oxidase–phenol aminophenazone (CHOD–PAP) method [33], and triglycerides by the glycerol phosphate oxidase–phenol aminophenazone (GPO–PAP) method [34]. Quality control sera were analyzed periodically to verify analytical accuracy and precision [33,35,36].

### 2.5. Colostrum and Milk Evaluation

Colostrum samples were collected individually from each ewe at 0, 24, and 48 h postpartum before lamb suckling during the morning feeding period. Milk samples were subsequently collected during the lactation period using the same sampling procedure. Samples were obtained before suckling to ensure consistency among animals.

Samples were immediately refrigerated at 4 °C, transported to the laboratory, and analyzed for chemical composition using a MilkoScan analyzer (Minor Type 78100, Foss Electric, Hillerød, Denmark). The concentrations of fat, protein, lactose, total solids (TS), and solids-not-fat (SNF) were determined and expressed as percentages (%). The instrument was calibrated before analysis according to the manufacturer’s recommendations [37].

Because colostrum composition plays a critical role in passive immunity transfer, neonatal survival, and early lamb growth, its nutritional composition was evaluated in relation to lamb performance traits [18].

### 2.6. Statistical Analysis

Data were analyzed using the GLM and MIXED procedures of SAS 9.4 [38] (SAS Institute Inc., Cary, NC, USA), as appropriate for the response variable. Lamb growth performance, serum biochemical variables, and colostrum and milk compositions measured over time were analyzed using repeated-measures mixed models. Overall lamb survival rates among treatments were compared using the Chi-square test. Statistical significance was declared at *p* < 0.05.

For repeated-measures analyses, several covariance structures were evaluated, and the structure with the lowest Akaike Information Criterion (AIC) and Bayesian Information Criterion (BIC) values was selected as the best-fitting model [39].

The statistical model was as follows:

Y_ijk_ = μ + τ_i_ + δ_ij_ + t_k_ +(τ * t)_ik_ + ε_ijk_; i = 1..., a; j = 1,...,b; k = 1, ..., *n*

where Y_ijk_ is the observed response variable, μ is the overall mean, τ_i_ is the fixed effect of dietary treatment (i = 1–4), t_k_ is the fixed effect of sampling time, (τ * t)_ik_ is the treatment × time interaction, δ_ij_ is the random effect of animal within treatment, and ε_ijk_ is the residual error, assumed to be normally distributed with a mean of zero and variance σ^2^.

The experimental unit for all measured variables was the individual animal (18 replicates per treatment), except for body condition score (BCS), which was evaluated in a representative subset of 12 ewes per treatment.

Because SFH inclusion levels represented a quantitative treatment factor (0, 12, 20, and 28% of dietary dry matter), orthogonal polynomial contrasts were used to evaluate linear and quadratic responses to increasing SFH inclusion. Contrast coefficients were generated in SAS using the actual dietary inclusion levels rather than assuming equal spacing among treatments. Linear and quadratic trends were estimated using the CONTRAST and ESTIMATE statements within the appropriate SAS procedures. When both linear and quadratic effects were significant (*p* ≤ 0.05), the quadratic response was considered to best describe the treatment pattern.

Simple linear regression (PROC REG, SAS) was used to evaluate the association between ewe BCS and lamb birth weight according to the following model:

Birth weight (kg) = β_0_ + β_1_ (BCS) + ε,

where β_0_ is the intercept, β_1_ is the regression slope, and ε is the random error term. Regression coefficients (intercept and slope) were estimated and tested against zero using Student’s *t*-tests. The coefficient of determination (R^2^), regression coefficients, corresponding *p*-values, and Pearson’s correlation coefficient (r = √R^2^) were obtained from the regression analysis. Statistical significance was declared at *p* < 0.05.

## 3. Results

### 3.1. Performance of Lambs

The growth performance of lambs born to dams fed graded levels of SFH is presented in Table 2. Maternal dietary treatment did not affect lamb birth weight (*p* = 0.991), with an overall mean of 4.29 kg. However, postnatal growth performance was influenced by dietary SFH inclusion. Weaning weight decreased linearly with increasing SFH levels (linear *p* = 0.044), declining from 28.03 kg in the control group to 25.11 kg in the S20 group. Average daily gain also showed a significant linear decrease in response to increasing SFH inclusion (linear *p* = 0.025), with lambs from SFH-supplemented ewes averaging 0.23 kg/day compared with 0.27 kg/day in the control group.

Body weight gain was affected by treatment (*p* = 0.04) and exhibited a significant quadratic response to increasing dietary SFH inclusion (quadratic *p* = 0.023), whereas the linear effect was not significant (linear *p* = 0.28). Lambs from the control group achieved the greatest body weight gain (23.68 kg), while those from the SFH treatments gained between 20.85 and 21.16 kg. The quadratic response indicates that the reduction in body weight gain was not proportional across SFH inclusion levels. Overall, maternal SFH supplementation had no effect on birth weight but was associated with reduced postnatal growth performance as dietary inclusion increased.

**Figure 1:**
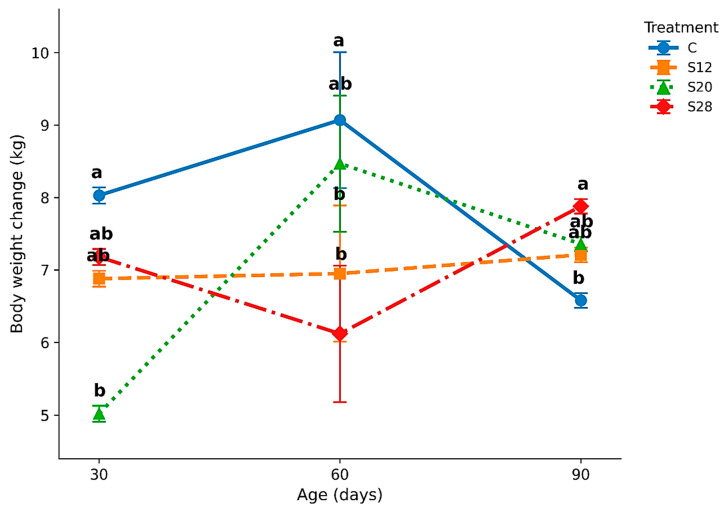
Changes in body weight of lambs born to dams fed experimental diets containing different levels of sunflower hulls. C = basal diet without sunflower hulls; S12, S20, and S28 = complete pelleted diets containing 12%, 20%, and 28% sunflower hulls, respectively. Different lowercase letters (a, ab, b) above data points within the same age indicate significant differences among treatments (*p* < 0.05); means sharing at least one common letter do not differ significantly.

The temporal pattern of lamb growth is presented in Figure 1. Body weight gain varied across the suckling period according to maternal dietary treatment. At 30 days of age, lambs from the control group exhibited the greatest body weight gain (8.03 kg), whereas the lowest gain was observed in the S20 group (5.02 kg). At 60 days, the control group had the highest gain (9.07 kg), followed by the S20 group (8.47 kg), whereas the S12 and S28 groups showed lower gains (6.95 and 6.12 kg, respectively). By 90 days of age, the pattern changed, with body weight gain declining in the control group (6.58 kg), whereas lambs from the SFH-supplemented groups exhibited greater gains, particularly in the S28 group (7.88 kg), followed by the S20 (7.36 kg) and S12 (7.21 kg) groups.

### 3.2. Relationship Between Ewe Body Condition Score and Lamb Birth Weight

**Figure 2:**
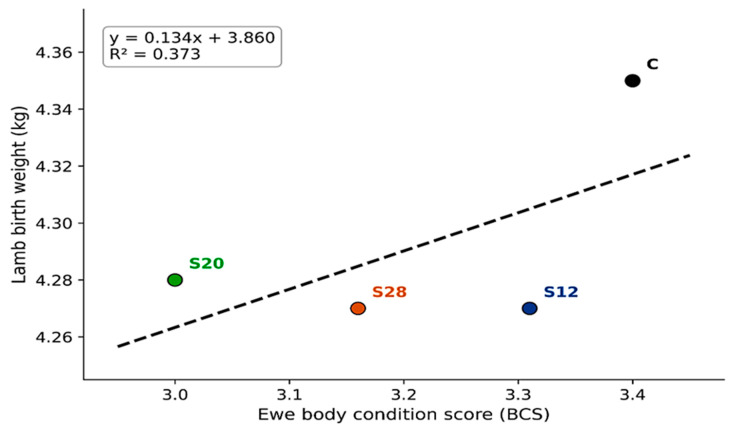
Relationship between ewe body condition score (BCS) and body weight of newborn lambs fed a complete diet with different levels of sunflower hulls (SFHs). C = basal diet without SFH; S12, S20, and S28 = diets containing 12%, 20%, and 28% SFH, respectively. Lamb birth weight increased with increasing ewe BCS (R^2^ = 0.37; r = 0.61; *p* = 0.04). The dashed line represents the least-squares linear regression fitted to individual ewe–lamb observations; points indicate treatment means.

The relationship between ewe BCS and lamb birth weight is illustrated in Figure 2. Because the figure displays treatment means for visualization purposes, whereas the regression analysis was conducted using individual ewe–lamb observations, the regression results are presented to describe the association between maternal BCS and lamb birth weight. Ewe BCS ranged from 3.00 to 3.40 across treatments, while lamb birth weight remained relatively consistent, varying from 4.27 to 4.35 kg.

Simple linear regression revealed a positive association between ewe BCS and lamb birth weight, with a regression coefficient significantly different from zero (β_1_ = 0.134, *p* = 0.04), indicating that lamb birth weight tended to increase with maternal BCS.

A significant positive Pearson correlation was also detected between ewe BCS and lamb birth weight (r = 0.61, *p* = 0.04). However, the coefficient of determination (R^2^ = 0.37) indicated that maternal BCS accounted for only 37% of the variation in lamb birth weight, suggesting that additional maternal, fetal, and environmental factors contributed to birth weight variation. Although ewes in the control group had the highest average BCS (3.40), the relatively small differences in BCS among treatments were not associated with meaningful differences in lamb birth weight. Overall, maternal BCS was positively associated with lamb birth weight, but its contribution to birth weight variation was moderate.

### 3.3. Colostrum Composition

The effect of dietary SFH inclusion on colostrum composition at 0, 24, and 48 h postpartum is presented in Table 3. At parturition (0 h), colostrum fat concentration was affected by treatment (*p* = 0.040) and exhibited a quadratic response to increasing SFH inclusion *(p* = 0.030). No treatment effects were detected for protein, lactose, or total solids concentrations at this time point (*p* > 0.05).

At 24 h postpartum, dietary treatment had no significant effect on colostrum components (*p* > 0.05). Similarly, no significant linear or quadratic responses were detected (*p* > 0.05). At 48 h postpartum, dietary treatment influenced colostrum fat (*p* = 0.032) and protein (*p* = 0.050) concentrations, both of which showed significant quadratic responses to increasing SFH inclusion (*p* = 0.041 and *p* = 0.022, respectively). In contrast, lactose and total solids concentrations were not affected by treatment or by linear or quadratic contrasts (*p* > 0.05). Overall, SFH supplementation influenced colostrum fat and protein concentrations at specific postpartum sampling times (*p* < 0.5), whereas lactose and total solids concentrations remained largely unaffected (*p* > 0.5).

### 3.4. Milk Composition

Milk composition data are presented in Table 4. Dietary treatment affected milk fat concentration (*p* = 0.012), with a significant quadratic response observed as the level of SFH inclusion increased (*p* = 0.022). Total solids concentration was also influenced by dietary treatment (*p* = 0.014) and exhibited a quadratic response to increasing SFH inclusion (*p* = 0.021). In contrast, milk protein and lactose concentrations were not affected by dietary treatment and showed no significant linear or quadratic responses (*p* > 0.05). Overall, dietary SFH inclusion altered milk fat and total solids concentrations, whereas milk protein and lactose concentrations remained stable across treatments.

### 3.5. Lamb Survival Rate

The survival rate of lambs from birth to weaning is presented in Table 5. At birth, survival rates ranged from 84.44% in S12 to 93.20% in S28, while the control group recorded a survival rate of 91.77%. A similar pattern was maintained throughout the suckling period. Lambs from the S12 and S20 groups consistently exhibited lower survival rates than those from the control and S28 groups. By weaning, the highest overall survival rate (*p* = 0.137) was observed in S28 (92.61%), followed by the control group (91.88%), whereas S12 (83.99%) and S20 (84.51%) showed lower survivability.

### 3.6. Blood Metabolites of Lambs

Serum biochemical parameters of lambs are presented in Table 6. Significant treatment × age interactions were detected for albumin and cholesterol concentrations (*p* < 0.05), indicating that the effects of maternal dietary SFH supplementation varied with lamb age. Age significantly affected serum albumin, cholesterol, triglyceride, and urea-N concentrations (*p* < 0.05), whereas treatment effects were limited to selected metabolites and sampling ages.

Dietary treatment affected serum glucose concentration at 30 days of age (*p* = 0.014), with a significant quadratic response to increasing dietary SFH inclusion (*p* = 0.041). However, glucose concentrations were not affected by treatment at 60 or 90 days of age or for the overall mean (*p* > 0.05).

Serum total protein concentration was affected by treatment only at 90 days of age (*p* = 0.015) and exhibited a significant quadratic response (*p* = 0.021). Similarly, albumin concentration was influenced by treatment at 90 days (*p* = 0.031), showing a significant linear response (*p* = 0.042).

Serum cholesterol concentration was affected by treatment at 30 days of age (*p* = 0.022) and showed a significant quadratic response (*p* = 0.020). No treatment effects were detected at 60 or 90 days of age or for the overall mean (*p* > 0.05).

Overall serum triglyceride concentration was affected by maternal dietary treatment (*p* = 0.041) and decreased linearly with increasing dietary SFH inclusion (*p* = 0.023), whereas no treatment effects were detected at individual sampling ages (*p* > 0.05). In contrast, serum urea-N, globulin, and NEFA concentrations were not affected by dietary treatment at any sampling age or for the overall mean (*p* > 0.05).

## 4. Discussion

The absence of treatment effects on lamb birth weight indicates that maternal supplementation with SFH during late gestation did not compromise fetal development [40]. In sheep, birth weight is generally maintained when maternal nutrient requirements are adequately met, even when dietary fiber sources differ [3,4,41,42]. Consistent with the present findings, previous studies have reported no significant changes in lamb birth weight when dietary fiber levels increased without reducing maternal energy and protein intake [43]. The relatively narrow range of ewe BCS (3.00–3.40) among treatments further suggests that maternal nutritional status remained adequate to support normal fetal growth throughout gestation. Although a positive association was observed between ewe body condition score and lamb birth weight, maternal condition remained within the recommended physiological range, which likely minimized treatment-related differences in fetal growth [3,44,45,46].

Although fetal growth was maintained, lambs born to SFH-fed ewes exhibited lower pre-weaning growth, suggesting that maternal nutrition during lactation rather than gestation was affected. SFHs contain high concentrations of lignified fiber, and increasing dietary inclusion increased dietary acid detergent fiber (ADF) and lignin concentrations, which may reduce nutrient digestibility and metabolizable energy supply [13,15,47]. Although digestibility, feed intake, milk yield, and energy balance were not measured, reduced nutrient availability for milk production and offspring growth represents a plausible explanation for the lower weaning weight and average daily gain. These findings are consistent with previous studies showing that excessive inclusion of high-fiber agricultural by-products may impair animal performance [22]. The growth pattern throughout the suckling period supports this interpretation. Early postnatal growth is strongly influenced by the quantity and quality of nutrients available to the neonate, particularly energy-rich milk components such as fat [48]. Lambs from SFH-fed ewes exhibited slower growth during early lactation, when milk is the primary nutrient source, whereas partial compensatory growth was evident later, particularly in the S28 group, as rumen development increased reliance on solid feed. Similar compensatory responses have been reported following periods of moderate nutritional restriction [15]. However, this adaptation was insufficient to overcome the reduction in overall pre-weaning performance.

A significant positive association was observed between ewe BCS and lamb birth weight (r = 0.61; β_1_ = 0.134, *p* = 0.04), although the relationship explained only a moderate proportion of the variation in birth weight. The relatively narrow BCS range among treatments (3.00–3.40), which remained within the recommended range for reproductive performance, likely contributed to the absence of treatment-related differences in lamb birth weight. Previous studies have shown that fetal growth is generally maintained through preferential maternal nutrient partitioning when ewe BCS remains within the optimal physiological range, whereas maternal condition has a greater influence on birth weight only under severe undernutrition or excessive body condition [3,44,45,46]. These findings suggest that, under the nutritional conditions of the present study, maternal BCS remained sufficient to support normal fetal development across all treatments.

Colostrum quality is a critical determinant of neonatal survival and early growth because it provides both nutrients and passive immune protection immediately after birth [49]. Moderate SFH inclusion altered selected colostrum components, particularly fat and protein concentrations. Increased colostrum fat may enhance neonatal energy supply and thermoregulation immediately after birth, whereas greater protein concentration may reflect altered mammary nutrient partitioning during the transition period [22]. In contrast, lactose remained unchanged, consistent with previous reports indicating that lactose synthesis is tightly and homeostatically regulated and relatively insensitive to dietary manipulation [18].

In the present study, milk fat and total solids decreased with increasing SFH inclusion, suggesting altered ruminal fermentation and reduced availability of acetate for *de novo* milk fat synthesis [12,13,50]. The physical and chemical characteristics of dietary fiber influence chewing activity, saliva secretion, rumen fermentation patterns, and the production of volatile fatty acids, particularly acetate, which serves as the primary precursor for *de novo* milk fat synthesis [50]. Increasing dietary concentrations of poorly digestible fiber, particularly ADF and lignin, may reduce nutrient digestibility and fermentation efficiency [12,13]. Although rumen fermentation and milk yield were not measured, the lower milk fat concentration may have reduced the energy available to suckling lambs and contributed to the reduced pre-weaning growth [51]. Milk protein and lactose remained unchanged, indicating that these components were less responsive to the dietary fiber source than milk fat [52].

Although lamb survival differed numerically among treatments, survival is influenced by multiple maternal, neonatal, and environmental factors [27]. Because colostrum immunoglobulin concentration, colostrum intake, maternal behavior, and causes of mortality were not evaluated, the biological basis for these differences cannot be determined and should be interpreted cautiously.

Blood biochemical parameters are widely used as indicators of nutritional status, metabolic adaptation, and health in growing lambs [53]. In the present study, most measured metabolites remained within normal physiological ranges, indicating that maternal SFH supplementation did not adversely affect lamb health [54]. This interpretation is consistent with the review by Braun et al. [19], who emphasized that physiological factors such as age, nutritional status, and developmental stage can substantially influence serum biochemical variables in sheep without necessarily indicating pathological conditions. Although significant treatment effects were detected for serum glucose, total protein, albumin, cholesterol, and triglyceride concentrations, these responses were limited to specific sampling ages or represented transient changes, suggesting normal metabolic adaptation rather than impaired physiological function.

The treatment effects observed for glucose and cholesterol at 30 days of age likely reflect differences in energy metabolism during the suckling period, when lambs rely primarily on milk as their main source of nutrients [54]. As lambs mature and progressively transition to solid feed, glucose metabolism undergoes physiological adaptation, resulting in age-related changes in circulating glucose concentrations [55,56]. Previous studies have demonstrated that milk nutrient intake directly influences carbohydrate and lipid metabolism in neonatal lambs [57,58]. Although maternal SFH supplementation affected these metabolites during early life, all measured values remained within the physiological reference intervals reported for healthy sheep [19].

Age-related increases in total protein and albumin concentrations are consistent with the maturation of the immune and digestive systems. Lower protein concentrations during early life likely reflect the transition from passive immunity acquired through colostrum to endogenous immunoglobulin synthesis [59,60]. As rumen development progresses, nitrogen utilization becomes more efficient, promoting increased albumin synthesis and circulating protein concentrations [61]. The treatment effects observed for total protein and albumin at 90 days, therefore, most likely reflect normal developmental changes in nutrient metabolism rather than adverse physiological responses, particularly because all values remained within established physiological reference ranges [19].

Serum lipid metabolites also exhibited age- and dietary treatment-related variation. Although cholesterol concentration was affected only at 30 days of age, this response was not maintained at later sampling ages, indicating a transient metabolic effect during early lactation. In contrast, the overall linear decline in triglyceride concentration with increasing maternal SFH inclusion may reflect modest changes in energy supply or lipid metabolism associated with the greater dietary fiber content of the maternal diets [22]. Similar age-related changes in serum lipid metabolites have been reported in growing lambs as milk consumption declines and rumen fermentation becomes the primary source of metabolic energy [55,56].

The absence of treatment effects on globulin, urea-N, and NEFA further indicates that maternal SFH supplementation did not adversely affect protein metabolism, nitrogen utilization, or lipid mobilization in the offspring. Overall, although maternal dietary SFH supplementation induced limited changes in selected biochemical variables, the maintenance of all measured metabolites within established physiological reference intervals supports the conclusion that the metabolic health of suckling lambs was not compromised [19,62].

The present findings should also be considered within the broader context of using alternative fibrous agro-industrial by-products in small ruminant nutrition. Previous studies have shown that by-products such as sugar beet pulp, soybean hulls, citrus pulp, palm kernel cake, olive cake, and wheat straw can partially replace conventional fiber sources without compromising animal performance when diets are properly formulated [63,64,65]. However, their nutritional value and optimal inclusion levels depend on fiber composition, lignification, digestibility, and their contribution to the overall nutrient supply. Similar to other lignocellulosic feed resources, SFHs provide an economical source of dietary fiber, although excessive inclusion may reduce diet quality because of their high lignin content and limited digestibility. Collectively, these findings highlight that the successful use of fibrous agricultural by-products depends on balancing dietary fiber requirements with adequate energy and nutrient availability to optimize animal performance while improving the sustainability of ruminant production systems [4,63,66,67]. Future studies should directly compare SFH with other fibrous agro-industrial by-products under equivalent nutritional conditions and evaluate nutrient digestibility, milk yield and composition, rumen fermentation characteristics, and economic efficiency to establish optimal feeding strategies for sustainable sheep production.

## 5. Conclusions

In conclusion, the present study demonstrates that SFH can be incorporated into the diets of Naemi ewes during late gestation and lactation as an alternative fiber source without adversely affecting lamb birth weight, survival, or overall metabolic health. However, the responses were dependent on the level of dietary inclusion, with higher SFH levels reducing pre-weaning lamb growth performance. These findings indicate that the sustainable use of SFH requires balancing the benefits of utilizing locally available agro-industrial by-products with the maintenance of animal productivity. Based on the productive and physiological responses observed, dietary inclusion of approximately 12–20% SFH (dry matter basis) appears to provide the most appropriate compromise between efficient by-product utilization and satisfactory offspring performance. Further studies should evaluate nutrient digestibility, milk yield and composition, rumen fermentation, and economic returns to refine optimal inclusion strategies under practical production conditions.

## Figures and Tables

**Table 1:** Ingredients and chemical composition of the experimental diets, % on a dry matter basis.

Ingredients (%)	C	S12	S20	S28
Yellow corn	32.56	20.41	22.21	29.20
Palm kernel meal	20.00	20.00	20.00	9.25
Soya hulls	16.30	16.33	6.51	
Sunflower meal	13.80	13.84	13.71	16.03
Sunflower hulls	0.00	12.00	20.00	28.00
Wheat bran	10.00	10.00	10.00	10.00
Molasses	5.00	5.00	5.00	5.00
Acid buffer	0.80	0.80	0.80	0.8
Limestone	0.72	0.74	0.87	0.90
Salt	0.52	0.56	0.57	0.49
Breeding premix	0.30	0.30	0.30	0.30
Chemical composition				
Dry Matter%	90.39	88.47	88.74	88.54
Protein%	14.6	15.7	13.3	14.1
Fiber%	18.26	20.78	21.81	22.16
Fat%	4.02	4.35	4.00	4.35
Cellulose%	19.77	21.35	18.51	21.06
Hemicellulose%	9.13	8.49	6.96	10.87
GE Kcal/Kg	3641	3613	3744	3710
ME (Mcal/kg DM)	2.40	2.35	2.37	2.34
ADF%	28.46	30.25	29.55	30.66
NDF%	37.59	38.74	36.50	41.52
Lignin%	7.37	6.99	8.88	9.07

GE: gross energy, ADF: acid detergent fiber, NDF: neutral detergent fiber. Metabolizable energy (ME) was estimated from total digestible nutrients (TDN) according to NRC equations. TDN (%) was calculated as 88.9 − (0.79 × ADF%), digestible energy (DE) as TDN × 0.04409, and ME as DE × 0.82. Energy values are expressed on a dry matter basis.

**Table 2:** Growth rate of lambs born to dams fed the experimental diets containing different levels of sunflower hulls.

Variables	Dietary Treatments ^1^				SEM	*p* Value	Contrast	
	C	S_12_	S_20_	S_28_			Linear	Quadratic
Birth weight, kg	4.35	4.27	4.28	4.27	0.19	0.991	0.492	0.346
Weaning weight, kg	28.03	25.31	25.11	25.43	0.59	0.050	0.044	0.182
BW change, kg	23.68	21.04	20.85	21.16	0.13	0.042	0.281	0.023
ADG, g/d	0.27	0.23	0.23	0.23	0.08	0.050	0.025	0.357

Treatment, linear, and quadratic effects of increasing dietary sunflower hull levels were evaluated by orthogonal polynomial contrasts, with significance declared at *p* < 0.05. ^1^ Values are for Naemi newborn (*n* = 18/treatment); C = the basal diet without sunflower supplementation; S12, S20, and S28 = complete pelleted diets supplemented with sunflower at levels of 12, 20, and 28%. SEM = standard error of means. BW, body weight; ADG, average daily gain.

**Table 3:** The effect of different levels of sunflower hulls on colostrum composition (%) of Naemi ewes.

Measurement, Unit	Dietary Treatments *				SEM	*p* Value	Contrast	
	C	S_12_	S_20_	S_28_			Linear	Quadratic
At birth								
Fat (%)	10.16	14.65	15.66	12.96	1.34	0.041	0.112	0.030
Protein (%)	11.92	11.60	13.40	12.14	0.86	0.513	0.299	0.112
Lactose (%)	2.81	1.90	1.60	2.40	0.48	0.367	0.371	0.192
Total solids (%)	26.61	27.80	31.53	27.90	2.55	0.570	0.522	0.211
At 24 postpartum								
Fat (%)	10.63	12.43	14.05	9.28	0.68	0.188	0.277	0.31
Protein (%)	9.75	10.75	12.57	10.82	0.28	0.471	0.621	0.23
Lactose (%)	3.27	2.15	1.91	2.92	0.20	0.100	0.290	0.16
Total solids (%)	25.67	27.40	30.58	26.71	0.25	0.161	0.323	0.16
At 48 postpartum								
Fat (%)	9.60	10.66	14.45	8.47	1.42	0.032	0.412	0.041
Protein (%)	6.42	10.61	11.53	9.85	1.10	0.049	0.031	0.022
Lactose (%)	3.85	2.60	2.00	3.26	0.46	0.101	0.212	0.111
Total solids (%)	22.33	24.15	30.78	22.12	2.15	0.112	0.178	0.232

* Treatment, linear, and quadratic effects of increasing dietary sunflower hull levels were evaluated by orthogonal polynomial contrasts, with significance declared at *p* < 0.05. *n* = 18 per treatment. C = the basal diet without sunflower supplementation; S12, S20, and S28 = complete pelleted diets supplemented with sunflower at levels of 12, 20, and 28%. SEM = standard error of means.

**Table 4:** The effect of different levels of sunflower hulls on milk composition (%) of Neami ewes.

Measurement, Unit	Dietary Treatments *				SEM	*p* Value	Contrast	
	C	S_12_	S_20_	S_28_			Linear	Quadratic
Fat (%)	8.25	5.72	6.16	6.54	0.43	0.012	0.030	0.022
Protein (%)	5.10	5.34	4.87	4.62	0.22	0.326	0.439	0.516
Lactose (%)	4.72	4.78	5.03	4.91	0.10	0.528	0.614	0.382
Total solids (%)	18.94	16.40	16.45	16.73	0.39	0.014	0.037	0.021

* Treatment, linear, and quadratic effects of increasing dietary sunflower hull levels were evaluated by orthogonal polynomial contrasts, with significance declared at *p* < 0.05. *n* = 18 per treatment; C = the basal diet without sunflower supplementation; S12, S20, and S28 = complete pelleted diets supplemented with sunflower at levels of 12, 20, and 28%. SEM = standard error of means.

**Table 5:** Survival rate (%) of lambs from birth to weaning as affected by maternal dietary sunflower hull supplementation.

Age, Day	Dietary Treatments				SEM	*p* Value
	C	S_12_	S_20_	S_28_		
0	91.77	84.44	84.77	93.20		
30	90.00	81.23	84.00	92.85		
60	94.25	85.60	85.40	93.00		
90	91.50	84.70	83.90	91.40		
Overall	91.88	83.99	84.51	92.61	2.68	0.137

C = the basal diet without sunflower supplementation; S12, S20, and S28 = complete pelleted diets supplemented with sunflower at levels of 12, 20, and 28%. Overall survival rates were compared among treatments using the Chi-square test. Differences were considered significant at *p* < 0.05. SEM = standard error of means. *n* = 18 per treatment.

**Table 6:** Effects of dietary sunflower hull supplementation on blood metabolite concentrations of Naemi lambs at different ages.

Measurement, Unit	Dietary Treatments *				SEM	*p* Value	Contrasts	
	C	S_12_	S_20_	S_28_			Linear	Quadratic
Glucose mg/dL								
30 d old	92.66	94.01	110.93	71.42	6.61	0.014	0.192	0.041
60 d old	81.87	83.61	88.51	97.43	3.91	0.580	0.714	0.360
90 d old	82.76	98.86	78.08	79.19	5.87	0.572	0.290	0.192
Overall	85.76	92.16	92.51	82.82	3.23	0.673	0.395	0.183
Total protein g/dL								
30 d old	4.22	4.76	4.29	6.70	0.68	0.532	0.141	0.224
60 d old	4.21	4.57	5.04	4.82	0.28	0.770	0.314	0.690
90 d old	4.76	6.43	5.27	5.47	0.20	0.015	0.102	0.021
Overall	4.43	5.25	4.90	5.66	0.25	0.381	0.190	0.183
Albumin g/dL								
30 d old	1.68	1.57	1.61	1.68	0.06	0.924	0.813	0.490
60 d old	1.66	1.66	1.64	1.60	0.05	0.970	0.090	0.793
90 d old	1.74	2.49	1.84	1.80	0.11	0.031	0.042	0.102
Overall	1.69	1.90	1.69	1.69	0.05	0.202 ^A,A*T^	0.114	0.321
Globulin g/dL								
30 d old	2.54	3.19	2.68	5.02	0.66	0.489	0.381	0.470
60 d old	2.55	2.91	3.39	3.21	0.27	0.722	0.072	0.672
90 d old	3.02	3.94	3.42	3.68	0.17	0.250	0.365	0.144
Overall	2.70	3.34	3.21	3.97	0.24	0.364	0.251	0.471
Cholesterol mg/dL								
30 d old	86.83	149.40	151.93	118.13	9.02	0.022	0.093	0.020
60 d old	116.30	82.95	81.93	96.36	6.38	0.182	0.912	0.113
90 d old	98.53	99.11	64.05	66.96	10.19	0.491	0.221	0.417
Overall	100.55	110.49	101.74	93.18	5.36	0.622 ^A,A*T^	0.134	0.162
Triglyceride’s mg/dL								
30 d old	95.40	96.87	91.45	72.43	6.02	0.464	0.322	0.410
60 d old	75.12	69.84	74.56	72.35	4.41	0.970	0.721	0.611
90 d old	73.15	58.64	55.36	58.82	3.31	0.233	0.188	0.135
Overall	81.22	75.11	72.21	60.81	2.81	0.041 ^A^	0.023	0.145
Urea- N mg/dL								
30 d old	13.85	13.24	13.03	15.48	0.87	0.781	0.612	0.52
60 d old	14.81	18.15	17.21	17.07	1.08	0.754	0.470	0.31
90 d old	20.14	22.11	20.35	19.71	0.95	0.840	0.713	0.32
Overall	16.26	17.83	16.49	17.95	0.64	0.562 ^A^	0.189	0.48
NEFA (mg/dL)								
30 d old	8.19	6.16	5.17	7.92	0.55	0.152	0.144	0.222
60 d old	5.04	5.22	4.54	6.07	0.54	0.830	0.590	0.434
90 d old	3.60	4.90	3.24	3.96	0.43	0.601	0.623	0.410
Overall	5.61	5.43	4.32	5.98	0.51	0.532	0.390	0.421

Treatment, linear, and quadratic effects of increasing dietary sunflower hull levels were evaluated by orthogonal polynomial contrasts, with significance declared at *p* < 0.05. *n* = 72; * C = the basal diet without sunflower supplementation; S12, S20, and S28 = complete pelleted diets supplemented with sunflower at levels of 12, 20, and 28%. SEM = standard error of means. A: Effect of age (*p* < 0.05); A*T: interaction between age and treatments (*p* < 0.05). *n* = 18 per treatment.

## Data Availability

The original contributions presented in this study are included in the article. Further inquiries can be directed to the corresponding author.
